# Variant Target Effects on Motor Learning With M1 Anodal tDCS

**DOI:** 10.1002/brb3.70743

**Published:** 2025-08-04

**Authors:** Joselyn Perez, Quinn McCallion, Brach Poston, Zachary A. Riley

**Affiliations:** ^1^ Department of Educational Psychology University of Connecticut Storrs Connecticut USA; ^2^ School of Health and Human Sciences Indiana University‐Indianapolis Indianapolis Indiana USA; ^3^ Department of Kinesiology and Nutrition Sciences University of Nevada‐Las Vegas Las Vegas Nevada USA; ^4^ Interdisciplinary Ph.D. Program in Neuroscience University of Nevada‐Las Vegas Las Vegas Nevada USA

## Abstract

**Purpose:**

Learning a motor skill usually involves practicing the same task repetitively with the same end target or goal. Many overhand throwing studies have documented accelerated learning when the task is practiced with the addition of anodal transcranial direct current stimulation (a‐tDCS) to the primary motor cortex (M1). However, these studies use the same target to throw at each time. The purpose of this study was to replicate previous work where a‐tDCS applied to M1 improved performance of a dart‐throwing task quicker than with SHAM stimulation, but to have subjects throw to different targets on the dartboard with each throw.

**Method:**

Sixty‐four healthy subjects practiced a dart‐throwing task with their non‐dominant arm. On the first visit, the subjects performed a pre‐ and post‐test, as well as a 20‐min practice in between, where they were randomized to receive 2 mA a‐tDCS over the contralateral M1 to the throwing hand (*n* = 33) or very brief stimulation (SHAM, *n* = 31). The subjects repeated testing 1 and 24 h later to test for retention.

**Finding:**

Both groups reduced their endpoint error and the variability in endpoint error (throwing consistency) over time. There were no significant differences between a‐tDCS and SHAM conditions in either of these measures.

**Conclusion:**

Unlike many previous studies, stimulation of M1 with a‐tDCS did not accelerate learning of a dart‐throwing task when the targets were constantly changed. It is hypothesized that this could have been due to the stimulation intensity being too high and possibly having a counter‐regulatory effect on cortical activity, or it could have been due to some other learning mechanism that requires more time and practice to develop when the endpoint or target of the task changes. Alternatively, this task may have benefited from stimulation of the cerebellum, where it is known that motor adaptation can efficiently update and improve movements.

## Graphical abstract

Subjects practiced throwing darts with their non‐dominant hand at random mini‐targets within the size of a traditional dartboard while getting M1 tDCS stimulation. All subjects improved, but stimulation did not lead to faster improvement, as was expected.

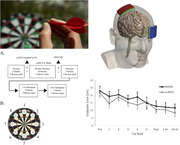

## 1 Introduction

Learning is a key component when individuals are trying to master physical tasks or skills. Motor learning is the process of obtaining and consolidating motor information, which is typically achieved through practice (Ammann et al. 2016; Karni et al. 1998; Nitsche, Schauenburg, et al. 2003; Reis et al. 2009). The region of the brain that has been studied most with motor learning is the primary motor cortex (M1), as this area is heavily involved in the execution of a movement (Ammann et al. 2016; Wang et al. 2021). With practice, structural and functional changes are occurring within the motor cortices, and these changes are responsible for the improvement of a task or skill (Reis and Fritsch 2011).

A non‐invasive brain stimulation technique that is commonly used to enhance motor learning is transcranial direct current stimulation (tDCS). tDCS can change neuronal excitability by altering resting membrane potential without suprathreshold activation of the targeted brain areas (Nitsche, Fricke, et al. 2003; Wiethoff et al. 2014). Specifically, anodal tDCS stimulation (a‐tDCS) produces an excitatory response in the targeted brain area by depolarizing the membrane potential to bring it closer to firing threshold. This can be driven by changes in the GABAergic function of the cells (Fritsch et al. 2010), long‐term potentiation (LTP) mechanisms (Spampinato and Celnik 2017), or even the indirect influence of glial cells (Gellner et al. 2016). The precise mechanisms depend on the area of the brain stimulated, task demands, the duration, and the intensity of the stimulation. Though many studies have shown that tDCS can accelerate motor skill learning or even help individuals attain proficiency beyond what they could with normal training, more research is needed to determine the ideal tDCS configuration and accompanying parameters (Ammann et al. 2016; Banissy and Muggleton 2013; Edwards et al. 2017).

Primarily, tDCS has been used to modulate motor learning when paired with a repeated motor task. The tasks most studied with tDCS of M1 focus on finger and hand dexterity movements, such as sequential finger tapping tasks and sequential visual isometric pinch force tasks (see Table 1 [Buch et al. 2017]). However, there have been a number of investigations into skilled whole‐arm movements such as reaching and throwing (Halakoo et al. 2020; Jackson et al. 2019; Mizuguchi et al. 2018; Pantovic et al. 2023; Scaramuzzi et al. 2025; Wang et al. 2021). The methodology across all these throwing studies is consistent with the movement always being directed toward the same target. For instance, Pantovic et al. (2023) required subjects to use an overhand throwing motion (like a baseball throw) to throw a tennis ball at a bullseye that was 6 m away. That study showed that the M1 tDCS group significantly reduced endpoint error over 3 days of practice. Meek et al. (2021) published a similar result with M1 tDCS, but with improved dart throwing at a bullseye over the duration of a single practice session.

**TABLE 1:** Displays the endpoint error (EE) in cm away from the target for the a‐tDCS and SHAM groups, for the four main time points.

	a‐tDCS	SHAM
Pre‐test EE (cm)	12.34 ± 0.62	12.59 ± 0.65
Post‐test EE (cm)	10.10 ± 0.49	10.57 ± 0.71
1‐h follow‐up EE (cm)	9.86 ± 0.44	10.50 ± 0.53
24‐h follow‐up EE (cm)	9.67 ± 0.40	10.40 ± 0.50

*Note*: Data are presented as mean ± standard error of mean.

In studies of complex tasks, such as overhand throwing, the start and end points can be approximately the same. Which means, even though whole body, multi‐joint coordination tasks are more transferrable to real‐world applications than a simple task (Cordo and Gurfinkel 2004; Wulf and Shea 2002), experimental procedures may be unrepresentative of activities of daily living, hobbies, or sports that are not repeatable in this manner. For example, a basketball player shoots from all over the floor, or a person reaches to their cabinets to put dishes away in different locations. These tasks, while similar in their general motion (e.g., shooting, throwing, reaching), require motor adaptive responses from sensory prediction errors to continually update the movements and continue motor learning (Hunter et al. 2009; Orban de Xivry and Shadmehr 2014).

Movements with changing endpoints (targets) also require reconsolidation, where the movement is still susceptible to being altered to include new information, even after it is encoded to memory (Censor et al. 2010). Reconsolidation in M1 can be made quicker with the addition of tDCS, leading to stable encoding of the motor memories (Sandrini et al. 2015). When performing many trials of a movement with variable targets and repeating the task up to 24 h later, it is expected that there would be a change in motor learning and reconsolidation caused by the application of tDCS on M1.

The purpose of the present study was to examine the effectiveness of a‐tDCS applied to the M1 while practicing a dart‐throwing task to different targets and compare the outcomes to a SHAM condition where tDCS was applied very briefly. It is expected that, because of motor adaptation, we should see continuous improvements in performance throughout the practice period in the first session, and that the improvements will be accelerated in the a‐tDCS condition. Furthermore, we expect that reconsolidation will be more effective in the a‐tDCS condition demonstrating how well the motor task was retained. This work is an extension of our previous work, where we showed a‐tDCS accelerating learning of a dart‐throwing task to a single bullseye target (Meek et al. 2021).

## 2 Materials and Methods

### 2.1 Participants

Sixty‐four healthy subjects (33 male; range: 18–28 years) participated in the study. The Indiana University Human Subjects IRB Committee approved the study (#1703811075), and all subjects provided written consent before participating. Recruitment for the study began on November 1, 2019 and did not finish until January 20, 2023 due to delays from the global pandemic. Subjects completed a checklist to self‐report that they had no neurological disorders, recent history of injury or disease involving the upper limbs, history of seizures or epilepsy, psychiatric disease, and no pacemaker or other metal implants in the upper body. Subjects also completed an Edinburgh Handedness Inventory (Oldfield 1971). Only three of the 64 subjects were identified as being left‐handed by the handedness inventory (EHI scores; right‐handed, 81.1 ± 3.42; left‐handed, −56.6 ± 14.52). Subjects completed the dart‐throwing task with their non‐dominant hand based on the outcome of the Edinburgh Handedness Inventory. The non‐dominant hand was chosen as the subjects had no experience throwing darts with that hand and likely had minimal cortical plasticity performing similar movements. The methodology used in the present study is similar to our previous study on dart‐throwing (Meek et al. 2021).

### 2.2 Experimental Timeline

**FIGURE 1:**
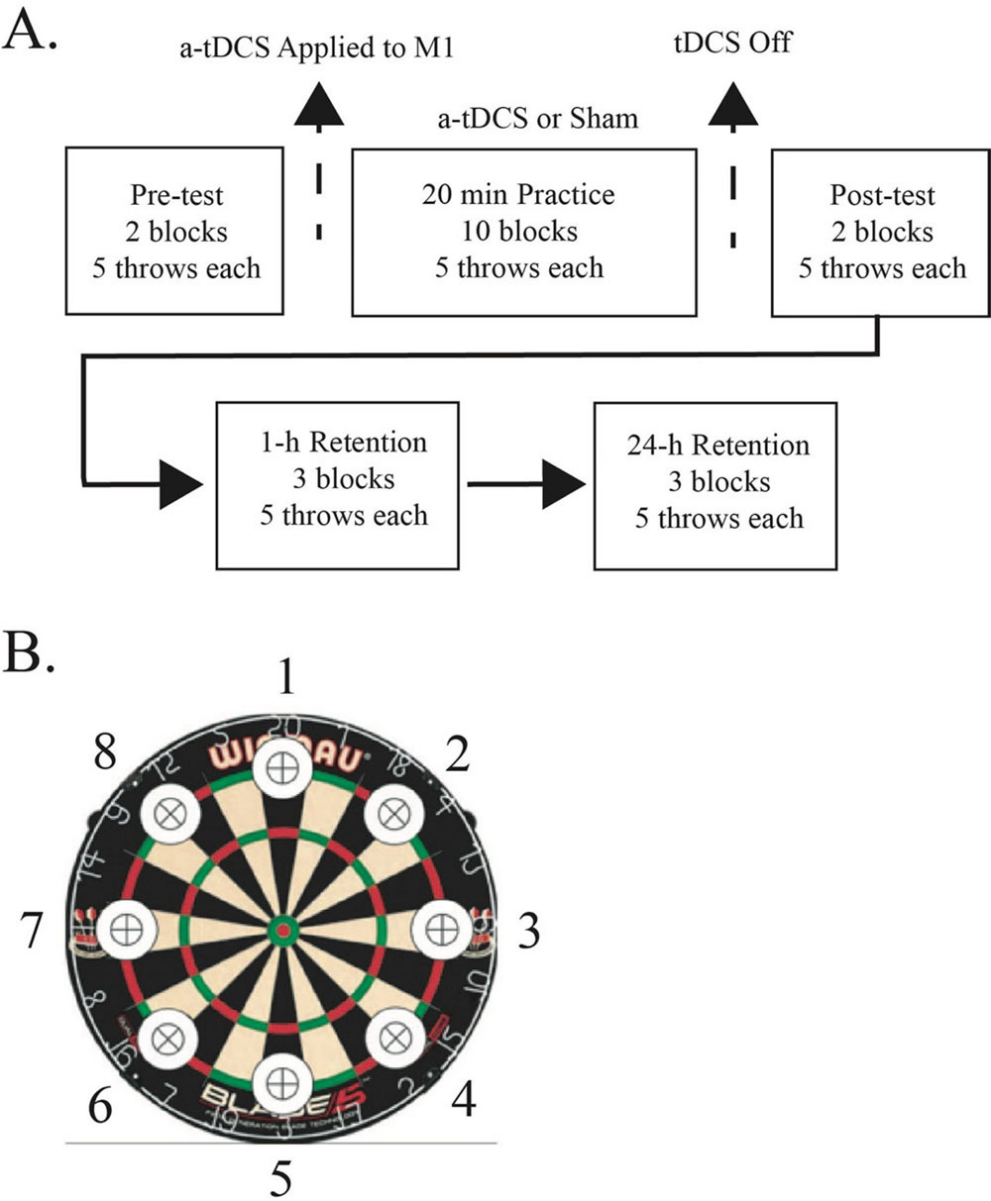
(A) Experimental timeline for the pre‐test, practice, and post‐test blocks of the study and the number of darts thrown in each block. (B) Picture of the dartboard that was used for the throwing task. The numbers around the perimeter of the board indicate the targets that were randomly selected each throw.

The subjects were randomly assigned to either SHAM (control; n = 31) or a‐tDCS (*n* = 33) stimulation groups based upon a randomization matrix created in MATLAB. They visited the lab on two occasions at approximately the same time of day (± 2 h). The experimental task involved subjects throwing darts at various targets with their non‐dominant hand. In total, each subject completed a pre‐test, practice period, post‐test, and two retention blocks (1 and 24 h follow‐up) of the dart‐throwing task, with the only difference being the stimulation condition (Figure 1A).

### 2.3 Variant Dart Task

Subjects stood behind a taped line that was 2.37 m from a Winmau Blade 4 Competition dartboard, and the board was mounted on a wall so that the center bullseye was 1.73 m from the floor, which is regulation distance for competitive dart throwing. All subjects used 23 g tungsten steel‐tipped darts. Subjects were given basic instructions on how to throw a dart and were given three familiarization throws. After the familiarization throws, the pre‐test required throwing at one of the eight different bullseye‐sized targets positioned evenly in a circle around the perimeter of the board (16.5 cm from each of these targets to the center bullseye; see Figure 1B). A random target was selected and announced to the subject right before each throw. A random number generator was used to determine what target (1–8) was thrown at, and the targets were not counterbalanced. Measurements were made on every throw with an inflexible metric ruler and endpoint error was quantified as the shortest distance between the selected target's center x–y coordinates and the dart's sticking point for each trial using the Pythagorean theorem (Jackson et al. 2019; Pantovic et al. 2023). Measurements were typed into a spreadsheet manually for each throw. A total of 10 throws (in two blocks with a brief rest between) were completed in the pre‐test. During practice, participants threw at one of the eight targets, randomly selected the same as the pre‐test. A total of 50 throws were completed during the practice period separated into 10 blocks, with approximately 50 s of rest between blocks (2 min blocks, total of 20 min). For the post‐test, subjects completed an additional 10 throws, the same as the pre‐test, at randomly selected targets. After the completion of the post‐test, subjects returned 1 and 24 h after the initial session to examine retention. In these retention sessions, subjects again threw at the random targets, with a total of 15 throws over three blocks.

### 2.4 Brain Stimulation

Participants were randomized to M1 a‐tDCS and SHAM stimulation groups. Both groups underwent the same preparation of the tDCS; however, the SHAM condition simulated the sensation of stimulation by incorporating a brief ramp‐up to 2 mA followed by a brief ramp‐down at the beginning and again at the end of the 20‐min session, with no current being delivered outside of the ramp times. None of the subjects had experienced tDCS prior to participating. Only one control subject reported that they thought they were in the stimulation condition, showing the effectiveness of the SHAM stimulation condition. For both groups, saline‐soaked sponges were placed on the scalp and stimulation was delivered with a Soterix 1 × 1 tDCS Device (Soterix Medical, NY). A 35 cm^2^ target electrode was fixed over the motor‐cortical position for the non‐dominant arm (C3 or C4, International 10–20 System; [Klem et al. 1999] and another 35 cm^2^ return electrode was placed on the ipsilateral supra‐orbital area (Fp1 and Fp2 according to the 10–20 EEG Coordinate System). All electrodes were secured with straps to ensure there was no movement during the throwing task. The current was delivered through the electrodes at a constant current of 2 mA for 20 min during the entire practice block of the dart‐throwing task. The stimulation intensity of 2 mA was chosen since it has been shown to provide the most reliable change in motor cortex excitability (Ammann et al. 2017).

### 2.5 Data Analysis

Endpoint error was averaged across 10 throws (two blocks) for the pre‐ and post‐tests. The practice period was averaged across every 10 throws (two blocks) as well, yielding five different time points. The two retention periods (1 and 24 h follow‐up) consisted of 15 throws over three blocks; however, the first block of five throws was discarded, and only the last 10 throws were averaged to be consistent with the other time points and exclude the initial effects of establishing their throwing technique again. The variability in endpoint error (standard deviation) was also calculated for each set of 10 throws, consistent with the time points listed above. Mixed‐model ANOVAs were used to compare the endpoint error and variability in endpoint error between the SHAM and a‐tDCS groups with repeated measures across all time points (within‐subjects: nine time points and between‐subjects: two groups [a‐tDCS & SHAM]). All analyses were conducted in SPSS v.29, and a significance level of *p* < 0.05 was used.

## 3 Results

**FIGURE 2:**
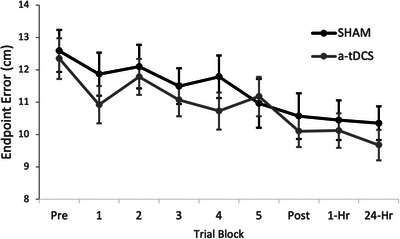
Group data showing the endpoint error (cm) across each of the trial blocks. Data are presented as means ± standard error.

Both the a‐tDCS and SHAM groups had a normal distribution of endpoint error in the pre‐test when examined with a Shapiro–Wilk test (*W* = 0.957, *p* = 0.249; and *W* = 0.972, *p* = 0.547, respectively*)*. An independent sample *t*‐test showed that there were no differences between the SHAM and a‐tDCS groups in the pre‐test endpoint error (*t*(62) = 0.267, *p* = 0.791). There was a significant main effect of time, showing that both groups reduced their endpoint error with practice (*F*(1,8) *=* 7.26, *p* ≤ 0.001, partial *η* ^2^ = 0.518, Figure 2). However, there was no main effect for stimulation condition (*F*(1,63) *=* 0.728, *p* = 0.389, partial *η* ^2^ = 0.012), and no interaction between time and stimulation condition (*F*(1,8) *=* 0.476, *p* = 0.826, partial *η* ^2^ = 0.008).

**FIGURE 3:**
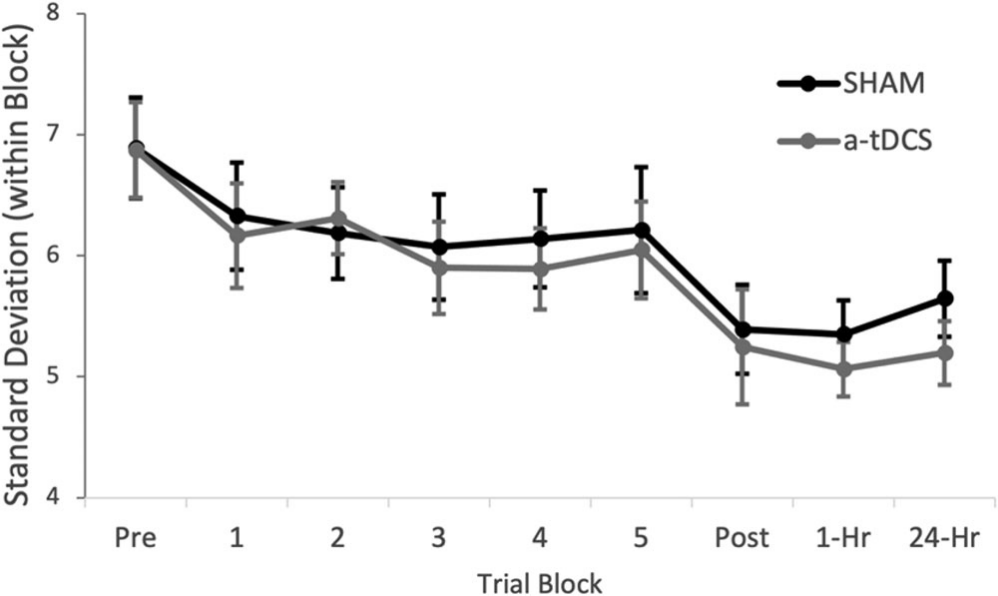
Group data showing the average standard deviation of the throws within each trial block. Data are presented as means ± standard error.

The results were similar in endpoint error variability, where there was less variability as time progressed (*F*(1,8) *=* 6.022, *p* ≤ 0.001, partial *η* ^2^ = 0.481, Figure 3), though it did not differ between groups (*F*(1,63) *=* 0.302, *p* = 0.585, partial *η* ^2^ = 0.005), and there was no interaction (*F*(1,8) *=* 0.210, *p* = 0.989, partial *η* ^2^ = 0.004).

## 4 Discussion

The purpose of the present study was to examine the effectiveness of a‐tDCS applied to the M1 while practicing a dart‐throwing task to different targets and compare the outcomes to a SHAM condition where no tDCS was applied. We expected that we would see continuous improvements throughout the first session due to the practice in both groups. This proved to be true; however, the second hypothesis, that the a‐tDCS would reduce endpoint error at a greater rate, was not observed. We could have analyzed the retention periods in a separate ANOVA model, though no differences would have been observed there either. There was a trend that the a‐tDCS group continued to improve during the retention periods, though it is unlikely this would have continued after 24 h since the subjects were not practicing the task anymore. Finally, both groups reduced their endpoint error variability, which is a measure of throw‐to‐throw consistency, though there were no differences between stimulation conditions. Modifying the task to throw at different targets on the same dartboard decreased the effectiveness of M1 a‐tDCS, which we had previously observed (Meek et al. 2021).

The expectation was that using 2 mA of current applied to M1 contralateral to the throwing hand would increase cortical excitability enough to improve the motor skill beyond what is expected under normal conditions. It has even been shown that 2 mA stimulation is a high enough intensity to produce reliable intra‐ and intersubject increases in M1 cortical excitability across multiple sessions, which is why it was chosen as the stimulation intensity in the present study (Ammann et al. 2017). However, a notable difference between the current study and our previous study where we found significant improvements with a‐tDCS of M1 in dart throwing (Meek et al. 2021) is that we had previously used 1 mA intensity. There are well‐documented intensity‐dependent effects with tDCS when using anywhere from 0.2 to 2 mA of current (Buch et al. 2017). For example, Lerner et al. (2021) reported that 2 mA of anodal current to M1 blocked improvements in a reaching task, even compared to a SHAM condition. Another study showed that 2 mA of *cathodal* tDCS to M1 actually increased cortical excitability (Mosayebi Samani et al. 2019), which is a surprising result because that current direction has been observed to reduce excitability and cerebral blood flow in the active tissues (Jamil et al. 2020; Lang et al. 2004). Instead of a linear dose‐response with tDCS current and cortical excitability, there is an alternative theory that higher stimulation intensities change LTP into long‐term depression (LTD) plasticity in the motor cortex, as a counter‐regulatory mechanism limiting excessive cortical activity (Elbert et al. 1981; Hassanzahraee et al. 2020).

An alternative explanation for the differences observed between Meek et al. 2021 and the present study could be in the framework of learning that this random target task required. In our previous study and many other throwing studies (Jackson et al. 2019; Mizuguchi et al. 2018; Pantovic et al. 2023; Scaramuzzi et al. 2025; Wang et al. 2021), a single target was used, which requires repetitive movements and leads to more stable motor memories in M1 (Mawase et al. 2017). While this can make learning faster due to less error‐based updating of an internal model (Diedrichsen et al. 2010), it also introduces bias to move the arm in the direction of the repeated movements (Huang et al. 2011; Verstynen and Sabes 2011). In our previous study, such a framework of learning would be enhanced with a‐tDCS, whereas in the present context of changing targets, it is likely that there was not enough practice or time for this to occur. Classen et al. (1998) showed that when changing the direction of a simple thumb movement, 15–30 min of continuous training was required to see changes in the stimulation‐evoked cortical networks. Our time scale of only 20 min of practice, combined with changing targets every throw, may have eliminated any effect tDCS had on the cortical region supporting motor learning.

The current application that we used in the current study was directed over the hand region of the motor cortex (C3 or C4, International 10–20 System). It is possible that the electrode size was insufficient to enable the spread of current to the arm and shoulder regions that may be more vital in a throwing task. However, this has been a consistent placement used in our and other researchers’ studies (Jackson et al. 2019; Meek et al. 2021; Mizuguchi et al. 2018; Pantovic et al. 2023; Scaramuzzi et al. 2025; Wilkins et al. 2024). It should be pointed out again that those previous studies performed throwing motions to a single target, which requires less kinematic variability than the current study. Consequently, even though throwing is a complex motor task, the motions of the shoulder, arm, and hand should be relatively consistent from throw‐to‐throw. Electrode placement higher on the scalp (medially toward CZ, International 10–20 System) could be more optimal in the present design, which would direct current more toward the arm and shoulder region of the motor cortex.

Alternatively, M1 may not have been the optimal stimulation site with the dart‐throwing task used in this study. The cerebellum, which is known to be a controller for motor learning (Morton and Bastian 2006), may have been a more appropriate given the ever‐changing targets the subjects were throwing at. In tasks with high amounts of variation, there is need for more continuous error feedback along cortico‐cerebellar tracts that will update internal models of the task (Butcher et al. 2017). Purkinje cells in the cerebellum send inhibitory outputs to the motor cortex to fine‐tune motor tasks and reconsolidate learning so that the appropriate feedforward predictions about the task can be made (Haruno et al. 2001; Izawa et al. 2012). For example, applying cathodal tDCS contralaterally on the cerebellum to the arm during a throwing task reduced its excitability and led to impaired motor learning in the proximal limb muscles (Weightman et al. 2021). In the same study, applying the same stimulation to the cerebellum during a joystick task with the hand, motor learning was increased, suggesting different pathways of excitatory and inhibitory tone onto M1 from the cerebellum. While the cerebellum may have been a better target for stimulation in the present study, past results show that increasing task complexity could impede the effectiveness of cerebellar tDCS (Weightman et al. 2021).

Transcranial direct current stimulation and other variations of non‐invasive brain stimulation have been used extensively as a tool for neuromodulation with motor skill learning and retention. The issue is that the promise once held for the invariant benefit of tDCS across all conditions is proving to be more nuanced. There are the obvious issues of what brain regions to target for different tasks. Once the brain region is selected, then there are issues such as inter‐ and intra‐subject variability, sham/blinding conditions, motor interference, and differences in the electrical current delivered (Horvath et al. 2014; Horvath et al. 2015). For every study that shows a reliable and reproducible neurophysiological effect on M1 from tDCS (Bashir et al. 2019), there is one that shows the opposite (Horvath et al. 2016). Then, to complicate interpretation even further, there could be an increase in excitability in M1, measured with transcranial magnetic stimulation, that is not even associated with the degree of motor learning (Pantovic et al. 2023). The only interpretation from this literature is that there may be no standardized best practices for tDCS (Buch et al. 2017) and that each task will demand a specialized tDCS application that will require exploration to determine the optimal parameters to use.

## 5 Conclusion

With a fixed target, performance in an overhand throwing task can potentially be improved at a faster rate with a‐tDCS applied to M1, though the results of that study are not robust due to the lack of a significant time × stimulation condition interaction (Meek et al. 2021). With a more variable overhand throwing task to different targets, in the current study, we demonstrated a‐tDCS of M1 does not significantly reduce endpoint error and improve performance. These results could be explained by the high intensity (2 mA) of stimulation used or the lack of practice time needed when the target of the task constantly changes. The site of stimulation used in the current study, over the hand area of M1, may have been less effective than if stimulation was shifted more over the arm and shoulder regions that produce the throwing motion. Alternatively, in other variable upper limb tasks, there is a strong cerebellar‐dependent process for the error adaptation and correction (Martin et al. 1996; Morton and Bastian 2006). Therefore, future studies utilizing variable upper limb tasks should either deliver less electrical current to M1 and use longer and more frequent practice periods, or just focus on the cerebellum as a target for tDCS. The results of the current study, combined with those of Meek et al. (2021), just show the caution that needs to be used when applying tDCS, as well as the care that needs to be taken in interpreting the findings.

## Author Contributions

**Joselyn Perez**: conceptualization, investigation, writing–original draft, methodology. **Quinn McCallion**: conceptualization, writing–original draft, writing–review and editing. **Brach Poston**: conceptualization, investigation, formal analysis, project administration, supervision. **Zachary A. Riley**: conceptualization, investigation; writing–original draft, methodology, validation, writing–review and editing, formal analysis, supervision.

## Peer Review

The peer review history for this article is available at https://publons.com/publon/10.1002/brb3.70743.

## Data Availability

The data that support the findings of this study are available from the corresponding author upon reasonable request.
